# Autosomal Dominant Intellectual Development Disorder-6 (MRD6) Without Seizures Linked to a De Novo Mutation in the grin2b Gene Revealed by Exome Sequencing: A Case Report of a Moroccan Child

**DOI:** 10.7759/cureus.46456

**Published:** 2023-10-04

**Authors:** Hinde El Mouhi, Meriame Abbassi, Hanane Sayel, Said Trhanint, Abdelhafid Natiq, Brahim El Hejjioui, Merym Jalte, Youssef Ahmadi, Sana Chaouki

**Affiliations:** 1 Laboratory of Biomedical and Translational Research, Faculty of Medicine and Pharmacy and Dental Medicine, Sidi Mohamed Ben Abdellah University, Fez, MAR; 2 Center for Doctoral Studies Engineering Sciences and Techniques, Faculty of Sciences and Technologies, Sidi Mohammed Ben Abdellah University, Fez, MAR; 3 Laboratory of Medical Genetics and Onco-genetics, Central Laboratory of Medical Analysis, Centre Hospitalier Universitaire (CHU) Hassan II, Fez, MAR; 4 Department of Genetics, Faculty of Medicine and Pharmacy, Mohammed V University, Rabat, MAR; 5 Laboratory of Biotechnology, Environment, Agri-food, and Health (LBEAH), Faculty of Sciences Dhar El Mahraz, Sidi Mohamed Ben Abdellah University, Fez, MAR; 6 Department of Pediatrics, Centre Hospitalier Universitaire (CHU) Hassan II, FEZ, MAR

**Keywords:** autosomal dominant intellectual development disorder-6, moroccan, de novo mutation, grin2b, exome sequencing, mrd6

## Abstract

Autosomal dominant intellectual development disorder-6 (MRD6) arises from a *grin2b *gene mutation, inducing neurodevelopmental issues. The effects of MRD6 encompass cognitive disabilities, seizures, muscle tone decline, and autism-like traits. Its severity ranges from mild impairment to severe epilepsy. The disorder's rarity is emphasized by roughly 100 reported GRIN2B-related cases, spotlighting the gene's significance in brain development.

We present the case of a three-year-old Moroccan boy who was referred to a neuropediatric department for a molecular diagnosis. Initial genetic testing yielded inconclusive results, and subsequent tests for Angelman syndrome and metabolic diseases showed no abnormalities. Given the complexity of the disorder, exome sequencing was employed to identify the underlying genetic cause.

Exome sequencing identified a nonsense (STOP) mutation c.3912C>G (p.Tyr1304Ter) in the *grin2b *gene in the heterozygous state known to be present in MRD6 (Online Mendelian Inheritance in Man (OMIM) 613970). The family segregation study shows that this is a de novo variant, which is confirmed by Sanger sequencing. This variant has not been previously reported in the GnomAD database. Based on current scientific knowledge, the variant is considered pathogenic (PVS1, PS2, PM2, PP3, PP5) according to the criteria of the American College of Medical Genetics and Genomics (ACMG). The mutation in the *grin2b *gene (p.Tyr1304Ter) was predicted to be deleterious through bioinformatics analysis tools.

This study highlights the crucial role of the *grin2b *gene in normal brain development and communication within the nervous system. It also sheds light on the impact of a novel genetic mutation, identified through exome sequencing, on causing an intellectual developmental disorder in a child patient from Morocco.

## Introduction

Intellectual disability (ID) and language disorders are neurodevelopmental conditions that arise in early childhood [1]. A neurodevelopmental disorder affects around 1%-3% of the global population. Genetic factors have a significant role in causing this condition, leading to limitations in both intellectual functioning and adaptive behavior from birth. The complexity of ID poses challenges in terms of both genetic and clinical diagnosis. However, the introduction of large-scale genome sequencing initiatives using a trio-based method has shown remarkable effectiveness. Nonetheless, there are still challenges in comprehending many genetic variations [2].

A combined strategy involving advanced techniques like next-generation sequencing, functional studies, electrophysiology, and bioinformatics analysis has unveiled fresh avenues for comprehending the underlying causes of ID. This approach not only helps in deciphering novel genes responsible for ID but also offers insights into their functional implications. This innovative approach provides potential targets for therapeutic interventions for ID and significantly enhances the accuracy and efficiency of diagnosing this condition.

The neurodevelopmental disorder associated with GRIN2B (glutamate ionotropic receptor N-methyl-D-aspartate (NMDA) type subunit 2B) is caused by mutations of the gene called *grin2b*, which provides instructions to make a protein called GluN2B, which is found in neurons and is primarily expressed during pre-birth development.

The GRIN2B is part of specialized protein structures called NMDA receptors, which play a role in normal brain development, and changes in the brain in response to experience (synaptic plasticity), learning, and memory [3].

Autosomal dominant intellectual development disorder-6 (MRD6) is a neurodevelopmental disorder caused by a genetic mutation in the *grin2b* gene. It is inherited in an autosomal dominant manner and affects the nervous system, leading to delays in cognitive and motor development as well as intellectual disability of varying severity. Additional symptoms can include seizures, low muscle tone, abnormal movements, and characteristics of autism. Many individuals with MRD6 may also have brain malformations visible on magnetic resonance imaging (MRI). The effects of the disorder can range from mild cognitive disability without seizures to severe brain disease, and the symptoms can vary widely between affected individuals [4].

The identification of the genes associated with intellectual developmental disorder represents a particular challenge in the absence of physical symptoms that guide researchers and clinicians toward a correct diagnosis [2]. Some mutations of the *grin2b* gene lead to the production of a non-functional GluN2B protein or prevent the production of any GluN2B protein from one copy of the gene in each cell. A shortage of this protein can reduce the number of functional NMDA receptors, which would decrease receptor activity in the cells [5]. Other mutations lead to the production of abnormal GluN2B proteins that likely alter the functioning of NMDA receptors; some mutations reduce NMDA receptor signaling while others increase it. Researchers and scientists currently lack a comprehensive understanding of the mechanisms through which abnormal receptor activity interferes with typical brain growth and development. Additionally, the reasons behind why both excessive and insufficient receptor activity leads to similar neurological issues in individuals with neurodevelopmental disorders linked to the *grin2b* gene remain unclear [6].

In this work, we report our observations about a Moroccan child referred to our neuropediatric service for the first time for psychomotor developmental and language delays. In view of the lack of clear clinical guidance and the complexity, phenotypic, and genetic heterogeneity of this group of diseases, we opted for exome sequencing as a method to enable genetic diagnosis in our patient. As a result, we identified a novel pathogenic causal mutation in the *grin2b* gene [7].

## Case presentation

Our patient is a three-year-old Moroccan boy who was admitted to the department of neuropediatrics at the Hassan II University Hospital in Fez, Morocco, for psychomotor retardation and absence of language without prior clinical diagnosis. He is the last child of a sibling group of three from a non-consanguineous marriage; his mother is 39 years old, and his father is 45 years old. He was born at term, and after an uneventful pregnancy and delivery, his birth weight was 3900g. No complications were noted after birth. He was seen for the first time at the age of 11 months because he could not sit without support. After that, he was able to sit by the age of one year and walk by two years, but he still could not climb the stairs or run. About language, he just pronounced the same syllables, having as an antecedent a maternal uncle with an undiagnosed intellectual development disorder who died at the age of 17 years following an accident.

The pediatric examination showed some signs of social interaction impairment, showing a mild autistic spectrum with manual stereotypies. The physical examination showed axial hypotonia and trunk ataxia, but no dysmorphic features (face and external genital organs). Since these clinical findings suggested neurological origin, a cerebral MRI, and an EEG were requested. The MRI showed a discrete T2 hypersignal and fluid-attenuated inversion recovery (FLAIR) of the right parietal subcortical white matter and a myelination defect, while the EEG was normal without any particularities. The search for Angelman syndrome and metabolic diseases came back negative too, with a normal karyotype. It led to the need for exome sequencing in response to the lack of diagnostic guidance. Informed parental consent for genetic testing was obtained. The clinical diagnosis and all detected variants are summarized (Table 1).

**Table 1:** The diagnosis of the patient with intellectual developmental disorder was made by the clinician. M: male; N: normal; AD: autosomal dominant

Age	3 years and 9 months
Age of onset of symptoms	11 months
Reason for first consultation	Delayed acquisition
Gender	M
Consanguinity	No
Delayed psychomotor and language development	Yes
EEG	N
Brain MRI	T2 hypersignal
Karyotype	46, XY
Gene/ Transcript	*grin2b */ NM_000834.5
Nucleotide change	c.3912C>G
Amino acid change	p.Tyr1304Ter
Legacy	AD
Origin of the variant	De novo

Exome sequencing and data analysis

Given the complexity, phenotypic, and genetic heterogeneity of intellectual developmental disorder and the absence of ‘hotspot’ mutations, we chose exome sequencing as a diagnostic strategy for our patient with this clinical entity.

The human genome exonic region enrichment system used is "TWIST Exome Custom CERBA V2" (TWIST), followed by a pair-end sequencing reaction (2x100bp) on a HiSeq1500 platform (Illumina). The data from this sequencing are analyzed using a computational pipeline as described by Thevenon et al. [8]. The average depth and quality threshold of the resulting sequence are determined for coding exons as well as splice sites of Online Mendelian Inheritance in Man (OMIM) protein-coding genes. The match between the trio was confirmed by microsatellite analysis.

A list to be interpreted is established according to the following criteria: (1) modifying a protein sequence and/or a canonical splice site; (2) with a frequency less than 1% according to the single nucleotide polymorphism database (dbSNP); (3) affecting a gene whose variations have previously been associated with human genetic pathology (OMIM). The interpretation of genetic variations consists of identifying the variations that are likely to be returned to the prescribing clinician after confirmation by an independent method. The interpretation of the "exome" analysis is restricted to the total of genes previously associated with human pathology (OMIM) and associated with the patient's pathology; this is determined by the prescribing clinician on an individual basis based on the clinical question posed. The identified variations are classified according to the international interpretation standards of the American College of Medical Genetics and Genomics (ACMG) [9].

Sanger analysis

Sanger sequencing was used to confirm the results of the exome analysis, and the National Center for Biotechnology Information (NCBI) Primer-Basic Local Alignment Search Tool (BLAST) tool was used to create primers for the detected mutations (Table 2).

**Table 2:** The primer pair used for Sanger sequencing Tm: melting temperature; %CG: CG base content in percent; GRIN2B-14F: forward primer of exon 14 of *grin2b* gene; GRIN2B-14F: reverse primer of exon 14 of *grin2b* gene

Primer name	Sequence	Size	%GC	Tm
GRIN2B-14F	ACCCTCAGAGCCCGACTAAT	20	55	60
GRIN2B-14R	ATCGGCCCTTGTCTTTCAGG	20	55	60

On 2% agarose gels, amplification products were electrophoresed. Utilizing dye terminator chemistry (ABI Prism® BigDye v3.1) and the Applied Biosystems Prism 3130 DNA Analyzer automated sequencer, Sanger sequencing was carried out. Using DNA variation analysis software (Sequence Scanner v1.0®), the resultant sequences were aligned with the reference genome (GRCh37/hg19).

Established variants were cross-referenced with the databases of the Human Gene Mutation Database (HGMD; http://www.biobaseinternational.com/product/HGMD), the Exome Variant Server, the 1000 Genomes Project, and the "ClinVar" database (http://www.ncbi.nlm.nih.gov/clinvar/).

Results

Exome sequencing (Exome Solo) identified a variation leading to a nonsense (STOP) mutation c.3912C>G (p.Tyr1304Ter) of the *grin2b* gene in a heterozygous state in the patient. This significant finding was further validated using Sanger sequencing, which confirmed the presence of the c.3912C>G (p.Tyr1304Ter) mutation.

The segregation study conducted revealed that this mutation was de novo, meaning it was not inherited from either of the parents. This finding supports the notion that the mutation occurred sporadically and is likely responsible for the patient's intellectual developmental disorder.

Furthermore, the identified variant, c.3912C>G (p.Tyr1304Ter), has not been previously reported in the GnomAD database, which suggests its rarity in the general population. According to the criteria of the ACMG, this variant is considered pathogenic based on multiple lines of evidence, including the presence of strong pathogenicity criteria (PVS1) and supporting moderate criteria such as strong conservation (PM2), absence from population databases (PP3), and in silico predictions (PS2, PP5).

To further assess the potential deleterious impact of the p.Tyr1304Ter mutation, various bioinformatics tools were employed. These tools, including BayesDel addAF, BayesDel noAF, PROVEAN, MutationTaster, PhyloP100, DANN, EIGEN, EIGEN PC, FATHMM-MKL, FATHMM-XF, and likelihood ratio test (LRT), collectively predicted the mutation to be deleterious. This bioinformatics analysis strengthens our confidence in the pathogenicity of the identified variant and its role in the patient's intellectual developmental disorder.

In summary, the identification of the c.3912C>G (p.Tyr1304Ter) mutation in the *grin2b* gene, both through exome sequencing and subsequent validation by Sanger sequencing, provides robust evidence of its involvement in the patient's clinical presentation. The rarity of this variant in population databases, along with its predicted deleterious impact, strongly supports its pathogenic nature. These findings contribute to our understanding of the genetic basis of intellectual developmental disorders and emphasize the importance of comprehensive genetic analyses in unraveling the underlying causes of such conditions.

## Discussion

The *grin2b *gene is associated with an autosomal dominant developmental disorder with or without seizures (OMIM 613970) or early childhood epilepsy-encephalopathy (OMIM 616139). All reported cases of neurodevelopmental disorders associated with the *grin2b* gene that were confirmed through molecular genetic testing in parents have been found to be caused by a new (de novo) pathogenic variant or deletion in the *grin2b* gene [5]. The exact prevalence of neurodevelopmental disorders caused by the *grin2b* gene is currently unknown, and the cases reported in medical literature to date are very rare. The prevalence of GRIN2B-related neurodevelopmental disorders among individuals with neurodevelopmental disorders and/or childhood-onset epilepsy is around 0.2% [5].

The penetrance of GRIN2B-related neurodevelopmental disorders is thought to be 100%. The *grin2b* gene plays a vital role in normal brain development, primarily in nerve cells, and is essential for learning and memory [3,10]. Genetic variations in the *grinb2* gene, which codes for the GluN2B subunit of the NMDA receptor, have been consistently associated with several neurological conditions, such as West syndrome, intellectual disability accompanied by focal epilepsy, developmental delay, macrocephaly, brain plasticity, infantile spasms, and Lennox-Gastaut syndrome. These conditions are characterized by abnormal brain development, seizures, cognitive impairment, and developmental delays [11].

The autosomal dominant intellectual developmental disorder associated with the *grin2b* gene (MRD6) is a rare neurological disorder, and less than 100 cases have been reported so far. A wide range of clinical and genetic features characterize the disorder, and two out of three cases are caused by genetic mutations. It is responsible for a significant lifelong disability and requires specialized care. The symptoms of this disorder include mild to profound developmental delay or intellectual disability in all affected individuals, as well as abnormalities of muscle tone (such as spasticity and/or hypotonia, sometimes associated with feeding difficulties), epilepsy, and autism spectrum disorders (ASD)/behavioral problems. Other common childhood findings include microcephaly, dystonic, dyskinetic, or choreiform movement disorder, and/or cortical visual impairment [5].

In this study, we performed exome sequencing on a Moroccan child with an intellectual developmental disorder without seizures of unknown origin. We detected a novel pathogenic de novo mutation in this patient; this mutation was identified in the *grin2b* gene, which encodes the Glun2B subunit of the NMDA receptor that plays an important role in pre-and post-natal neurodevelopment as well as in neurotransmission.

The *grin2b* gene is located on chromosome 12p13, contains 27 exons, and codes for the GluN2B subunit of the NMDA receptor. This receptor is highly expressed prenatally; however, its expression decreases after birth in most brain regions [10]. During late embryogenesis and early postnatal development, GluN2B expression is high and plays a critical role in rapid cortical synaptogenesis. Studies on mice that lack the* grin2b* gene have shown neonatal death while increasing the expression of *grin2b* in the forebrain of mice improved long-term potentiation and spatial memory [5].

The NMDA receptor is a transmembrane ionotropic glutamate receptor that plays a key role in synaptic plasticity, learning, and memory. It is composed of four subunits, two of which are GluN1 subunits, and the other two are GluN2 subunits. There are four types of GluN2 subunits: GluN2A, GluN2B, GluN2C, and GluN2D. The subunit composition of the NMDA receptor affects its biophysical properties, pharmacology, and subcellular localization.

The GluN1 subunit is essential for the formation of a functional NMDA receptor and binds to glycine, while the GluN2 subunit binds to glutamate. The GluN2B subunit has a distinct role in NMDA receptor function due to its unique brain distribution and prolonged activation, which contribute to synaptic plasticity, learning, and memory. The GluN2B subunit is highly expressed during development and in specific brain regions such as the amygdala and prefrontal cortex hippocampus [12]. The NMDA receptor complexes are made up of different subunits that form ion channels. These channels are activated by glutamate and glycine binding and by membrane depolarization. The subunit composition affects the sensitivity to glutamate and the kinetics of the channels. At extrasynaptic sites, the NMDA receptors, along with death-associated protein kinase 1 (DAPK1), contribute to stroke damage. When the NMDA receptor is phosphorylated by DAPK1, it results in increased channel activity and a harmful influx of calcium ions, leading to irreversible neuronal death. The NMDA receptor also plays a role in neural pattern formation during brain development and in long-term depression of hippocampus membrane currents, as well as synaptic plasticity [12].

The NMDA receptors, which include GluN2B, play a crucial role in excitatory neurotransmission in the mammalian brain by binding to glutamate and forming highly permeable calcium channels that are blocked by extracellular magnesium in a voltage-dependent manner. Mutations in the *grin2b* gene can disrupt these functions, leading to various neurodevelopmental diseases [13].

The GluN2B receptors are a protein of 1484 amino acids distributed over several domains (Table 3) [14].

**Table 3:** The NMDA 2B protein binding domains, regions, and ligands A: alanine; R: arginine; N: asparagine; D: aspartate; C: cysteine; Q: glutamine; E: glutamate; G: glycine; H: histidine; I: isoleucine; L: leucine; K: lysine; M: methionine; F: phenylalanine; P: proline; S: serine; T: threonine; W: tryptophan; Y: tyrosine; V: valine; Zn: zinc

Domain	Position	Sequence (AA)	Ligand	
Binding site	127	H	Zn2+	
Binding site	284	E	Zn2+	
Binding site	514	T	L-glutamate	
Binding site	519	R	L-glutamate	
Region	604-623	KAIWLLWGLVFNNSVPVQNP	Pore-forming	
Site	615	N	Functional determinant of NMDA receptors	
Binding site	690-691	ST	L-glutamate	
Binding site	732	D	L-glutamate	
Region	1074-1097	EGNAAKRRKQQYKDSLKKRPASAK	Disordered	
Region	1162-1194	FKRDSVSGGGPCTNRSHLKHGAGDKHGVVSGVP	Disordered	
Region	1269-1302	PVAVPSNAPSTKYPQSPTNSKAQKKTRNKLRRQH	Disordered	
Region	1292-1304	KKTRNKLRRQHSY	Interaction with DAPK1	
Motif	1483-1485	SDV	PDZ-binding	

They're a major subtype of glutamate receptors at extrasynaptic sites that link multiple intracellular catabolic processes responsible for irreversible neuronal death.

The GluN2B subunit physically and functionally interacts with DAPK1 at extra-synaptic sites [15]. The DAPK1 directly binds with the GluN2B subunit receptor C-terminal tail, consisting of amino acids 1290-1310 (GluN2B(CT)). A constitutively active DAPK1 phosphorylates the GluN2B subunit at Ser-1303 and, in turn, enhances the NR1/NR2B receptor channel conductance.

**Figure 1:**
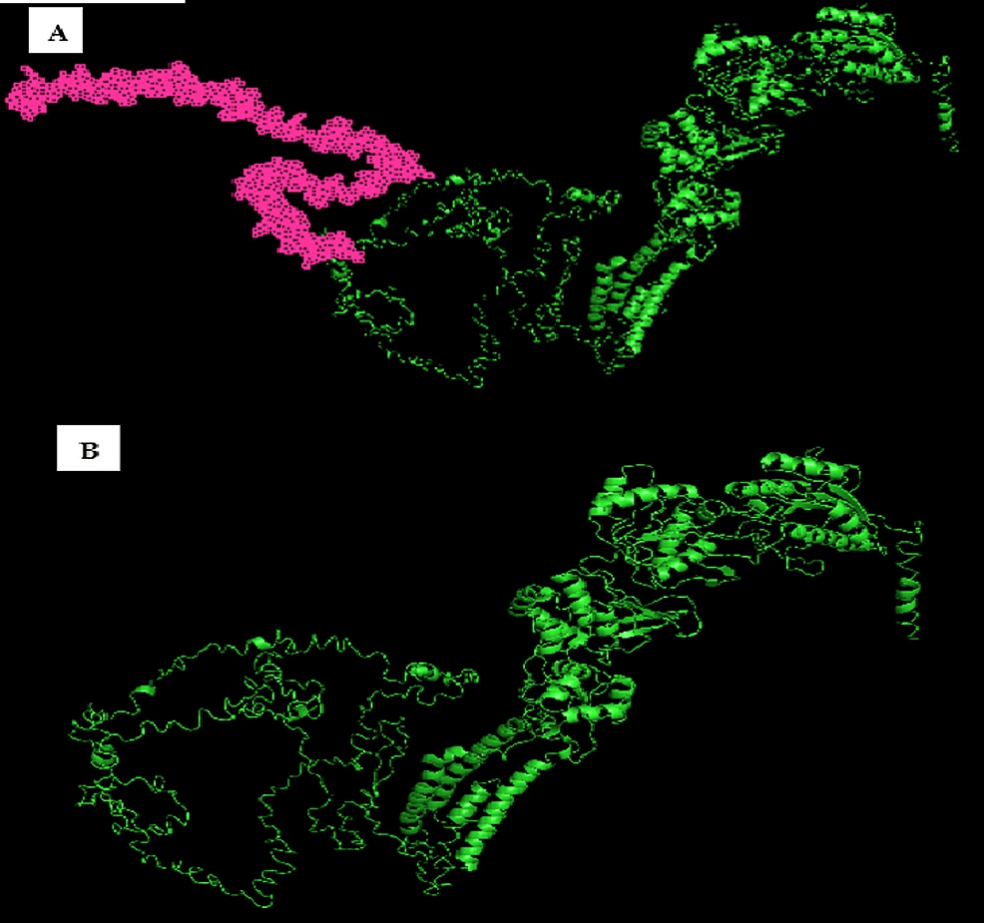
The 3D prediction of GRIN2B protein: (A) wild-type protein; (B) mutated GRIN2B protein. The prediction of the 3D structure of the GRIN2B protein, both wild-type and mutated, is created and predicted using the bioinformatic tool I-Tasser.

The c.3912C>G mutation in the *grin2b* gene found in our patient results in the production of a truncated p.Tyr1304Ter protein. This mutation deletes the sequence from residue 1304 at the end of the protein to residue 1484 (Figure 1), which affects the ability of DAPK1 to phosphorylate the GluN2B receptor and disrupts the regulation of its activity as well as glutamate neurotransmission from the NMDA receptor.

These effects have several potential consequences, including alteration of the receptor's ionic conductance, alteration of its localization in the postsynaptic membrane, and disruption of normal neuronal signaling associated with the NMDA receptor. This could potentially affect synaptic plasticity and cognitive function, which explains the impaired intellectual development phenotype found in the patient.

The DAPK1 directly binds with the GluN2B receptor C-terminal tail, consisting of amino acids 1290-1310 (NR2BCT) [16]. The p.Tyr1304Ter (GRIN2B) mutation in the NMDA receptor that eliminates the binding domain with DAPK1 can affect NMDA signaling and regulation of apoptosis. The binding domain between the GluN2B receptor and DAPK1 is crucial for the stability and activation of DAPK1. If this domain is eliminated by a mutation, it can result in reduced binding between the GluN2B receptor and DAPK1, which can reduce the activation of DAPK1. This can also affect NMDA signaling, which can disrupt neurotransmission and neuronal plasticity. Deregulation of apoptosis and NMDA signaling can contribute to neurodegenerative, neuropsychiatric, and immune system disorders.

In addition, and to strengthen our study, an in silico bioinformatics analysis of the mutated protein was performed, which highlighted that the p.Tyr1304Ter mutation in the GRIN2B protein is predicted to be deleterious using the following tools: MutationTaster, PhyloP100, PROVEAN, LRT, BayesDel addAF, BayesDel noAF, DANN, EIGEN, and FATHMM.

The scores obtained from these tools were analyzed as follows:

A MutationTaster score of 1 is a prediction that a genetic variant is probably damaging to the protein encoded by the affected gene. MutationTaster is a computational tool that uses a variety of data sources, such as population genetics data, functional data, and sequence information, to predict the impact of genetic variants on protein function.

The MutationTaster score ranges from 0 to one, with higher scores indicating a higher likelihood that the variant is deleterious. A score of 1 is at the high end of this range, indicating a strong prediction of pathogenicity.

A PROVEAN score is a numerical value used to evaluate the significance of a variant in a specific gene. This score is a predictive sustainability score ranging from -8.652 to 2.0, with negative values indicating a tendency to be harmful and positive values indicating a tendency to be benign.

A value of -8.652 is very negative and suggests that the variant in question is highly harmful and is likely to cause a negative effect on the protein encoded by the gene.

The PhyloP100 tool is a conservation-based method used in computational biology to predict the functional impact of genetic variants. The PhyloP100 gene revealed a score of -0.051, which indicates that the mutation in question is conserved in species evolution and that the protein is considered to be functionally important.

A score in the BayesDel addAF and BayesDel noAF tools indicates the predicted deleteriousness of a mutation in a human gene. In BayesDel addAF and BayesDel noAF, the pathogenicity score ranges from 0 to 1, with higher scores indicating a higher likelihood of the mutation being disease-causing. The Bayes-Del addAF revealed a score of 0.625.

A score of 0.625 would suggest that the mutation is considered to have a moderate effect on gene and protein function.

A score of 0.66 revealed by the BayesDel noAF tool would suggest that the mutation is considered to have a moderate to high effect on gene and protein function.

The likelihood ratio test (LRT) predicts deleterious variants through the identification of highly conserved amino acid regions using a comparative genomics data set of 32 vertebrate species; ranging: from 0 to one. The LRT score obtained revealed that the variant obtained is deleterious.

By analyzing the patient's clinical information, it was confirmed that his symptoms were consistent with the described disorder. Based on this, the identified genetic variation (p.Tyr1304Ter in the *grin2b* gene) is considered to be the cause of the symptoms observed in the patient.

Variant class and intellectual outcome show a significant correlation: heterozygotes for a *grin2b* pathogenic variant resulting in a null allele (e.g., nonsense or frameshift variants, deletion involving whole exons or the entire gene, translocation and inversion disrupting *grin2b*) tended to display mild or moderate intellectual disability (ID), while heterozygotes for pathogenic missense variants displayed severe ID (Fisher's exact test, p=0.0079) [5], which explains a moderate clinical picture in our patient.

Since the identification of the first pathogenic mutations in NMDA receptors [17,18], more than 60 *grin2b* variants have been reported in the literature and identified in individuals with a variety of neurodevelopmental disorders, including ID, developmental delay (DD), ASD, epileptic encephalopathy (EE), schizophrenia (SCZ), and, to a lesser extent, attention deficit hyperactivity disorder (ADHD), cerebral visual impairment (CVI), and Alzheimer's disease (AD) [10]. This highlights the important role of the *grin2b* gene in normal brain development and its potential impact on various neurological conditions.

Overall, the use of exome sequencing is a powerful approach for the molecular diagnosis of heterogeneous neurological disorders such as autosomal dominant intellectual development disorder in clinical practice. It has a high diagnostic yield and can be used to identify mutations, establish a definitive diagnosis, tailor appropriate medications, predict prognosis, and provide genetic counseling to families. Genetic counseling is important to inform parents of the risk of recurrence and the possibility of an early prenatal diagnosis. As more clinicians use exome sequencing in their practice, we can expect to see an increase in the discovery of rare variants and de novo mutations in patients with neurodevelopmental disorders, which will aid in the understanding and management of these conditions.

## Conclusions

This article describes a study in which exome analysis was performed on a Moroccan child with an intellectual developmental disorder of unknown origin. The results revealed a de novo pathogenic mutation in the *grin2b* gene, which codes for the Glun2B subunit of the NMDA receptor. Mutations in this gene have been associated with various neurodevelopmental disorders, including ID, DD, ASD, and epilepsy.

The use of exome sequencing is a powerful approach for the molecular diagnosis of these disorders, with a high diagnostic yield. This allows for a more efficient and accurate diagnosis and provides valuable information for treatment, management, and genetic counseling for the patient and their family. In this case, the c.3912C>G (p.Tyr1304Ter) mutation in the *grin2b* gene confirmed the diagnosis of an autosomal dominant intellectual development disorder, and genetic counseling was provided to inform the parents of the risk of recurrence in a future pregnancy and the possibility of early prenatal diagnosis.
